# Transboundary Animal Diseases Associated With Cross‐Border Camel Movement. A Systematic Review and Meta‐Analysis

**DOI:** 10.1155/tbed/6650796

**Published:** 2026-02-27

**Authors:** Alex A. Adikwu, Theophilus I. Emeto, Paul F. Horwood, Olajide A. Owolodun, Andrew M. Adamu, Oyelola A. Adegboye

**Affiliations:** ^1^ Department of Public Health and Tropical Medicine, College of Medicine and Dentistry, James Cook University, Townsville, Queensland, Australia, jcu.edu.au; ^2^ Department of Public Health and Preventive Medicine, College of Veterinary Medicine, University of Agriculture, Makurdi, Nigeria, uam.edu.ng; ^3^ Centre for Tropical Biosecurity, James Cook University, Townsville and Cairns, Queensland, Australia, jcu.edu.au; ^4^ Australian Institute of Tropical Health and Medicine, James Cook University, Townsville and Cairns, Queensland, 4811, Australia, jcu.edu.au; ^5^ World Health Organisation Collaborating Centre for Vector-Borne and Neglected Tropical Diseases, College of Medicine and Dentistry, James Cook University, Townsville, Queensland, 4811, Australia, jcu.edu.au; ^6^ Department of Veterinary Science, College of Science and Engineering, James Cook University, Townsville and Cairns, Queensland, Australia, jcu.edu.au; ^7^ Biotechnology Division, National Veterinary Research Institute, Vom, 930001, Nigeria, nvri.gov.ng; ^8^ Menzies School of Health Research, Charles Darwin University, Darwin, Northen Territory, 0810, Australia, cdu.edu.au

**Keywords:** camel, cross-border, diseases, meta-analysis, prevalence, transboundary, zoonosis

## Abstract

Transboundary animal diseases (TADs) are contagious diseases that significantly impact livestock health, public health and economic stability. In regions with frequent cross‐border trade and transhumance involving camels, particularly the Middle East and Africa, TADs pose a heightened One Health risk due to their zoonotic potential and capacity to spread rapidly across national boundaries. This review synthesises current knowledge on TADs, with a focus on zoonotic pathogens affecting camels involved in cross‐border movement, identifies geographical trends, and highlights research gaps to inform surveillance and control strategies. We conducted a comprehensive search across Ovid Medline, PubMed, Web of Science, Scopus, and Cochrane databases without time restrictions. Eligible studies were assessed for study quality and risk of bias using the Joanna Briggs Institute Critical Appraisal Checklist tools. Pooled prevalence estimates for TADs were calculated using random‐effects models, with subgroup and meta‐regression analyses to explore heterogeneity. Forty‐five eligible articles were included, identifying 15 zoonotic TADs. Middle East respiratory syndrome (MERS) (34%), Rift Valley fever (RVF) (15%). The cross‐border movements of camels contribute to the transnational spread of TADs, exacerbated by informal trade routes and nomadic pastoralism in arid regions. Our findings highlight the urgent need for harmonised surveillance and control strategies to mitigate the spread of zoonotic TADs through camel trade. Therefore, strengthening cross‐border surveillance, harmonising diagnostic protocols, and integrating animal‐human‐environment data within a One Health framework is critical to mitigating zoonotic disease threats in these regions.

## 1. Introduction

Transboundary animal diseases (TADs) are contagious diseases that are detrimental to the economy of countries; they can quickly spread to other nations, escalating into epidemic proportions, necessitating international cooperation for control and eradication [1, 2]. The Food and Agriculture Organization [3, 4] and World Organization for Animal Health (WOAH) [5] list of emerging TADs includes Middle East respiratory syndrome (MERS), severe acute respiratory syndrome (SARS), highly pathogenic avian influenza (HPAI), West Nile fever (WNF), Rift Valley fever (RVF), Crimean Congo haemorrhagic fever (CCHF), lumpy skin disease (LSD), bovine spongiform encephalitis (mad cow disease caused by prion), African horse sickness, bluetongue (BT), *peste des petits* ruminant (PPR), foot‐and‐mouth disease (FMD), African swine fever (ASF), contagious bovine pleuropneumonia (CBPP), Hendra, Nipah, Ebola, and Zika virus diseases [6–8].

TADs have the potential to endanger the world’s food production and supply through impacts to animal production, trade barriers that limit the movement of live animals and animal products, production deficiencies brought on by the loss of animal power, or even through a decrease in human productivity in the case of zoonoses [9–11]. Although attention is primarily focused on domestic livestock such as cattle, sheep, goats, and companion animals like dogs and cats, recently there has been growing attention on camels [12, 13], donkeys, and horses due to their increasing role in TAD transmission dynamics [14, 15].

Old‐World camelids, including the Bactrian camel (*Camelus bactrianus*) and dromedary camel (*Camelus dromedarius*), as well as New‐World camelids such as the alpaca (*Vicugna pacos*) and llama (*Lama glama*), can serve as potential sources of infectious diseases [16–18]. Camelids are generally known for their adaptive features and resilience to extremely harsh environments and climatic conditions [19]. These capabilities have created the perception that camels are resistant to infectious diseases commonly affecting other domestic animals, resulting in them receiving little or no veterinary attention. However, research has now shown that camels are sensitive to various infectious diseases and may potentially be reservoirs for pathogens of veterinary and public health importance [20].

Globally, there are ~30 million dromedary camels, predominantly distributed across the Middle East and Africa [21–23]. Although considered wild, the population of camels in Australia is estimated at 400,000, with a significant portion destined for export to national and international markets for meat and milk production [24, 25]. In Europe and the USA, camel populations are relatively small, with an estimated 5,000 and 3,000 animals, respectively. Nevertheless, information regarding trans‐border movement, commercial activities, and camel disease studies remains limited and largely undocumented in many regions of the world [26].

Over the years, camel migration, international trade, and trans‐border movements of camels and camel products have significantly increased the transmission and spread of TADs [24, 27]. For instance, a study carried out in Saudi Arabia found that 86.3% of 1257 imported camels, primarily from Sudan and Djibouti, were seropositive for MERS [28]. Another study detected brucellosis in camels imported into Ethiopia [29]. These findings can be linked to the cross‐border camel trade from the Greater Horn of Africa (GHA) to Middle Eastern countries, which represents the largest global camel market [27], with an estimated 250,000 to 300,000 camels exported annually from Sudan and Somalia [30, 31]. Furthermore, the Birqash market in Egypt (the largest market in the GHA) receives camels from Eritrea, Ethiopia, Somalia, Kenya, and Sudan [32]. In addition to the import and export of camels in the Sahelian region, unrestricted transhumance routes and drought caused by changing climate conditions are contributory factors in TAD spread [33, 34].

Although camels can facilitate cross‐border transmission and pose zoonotic risks, global data on the prevalence of TADs in transported camels remains limited. Therefore, to inform surveillance and control strategies, this review aimed to (i) synthesise current knowledge on TADs, with a focus on zoonotic pathogens affecting camels involved in cross‐border movement, (ii) identify the research gaps, and (iii) compile data on the prevalence and risk factors of these pathogens in camels involved in international trade and transhumance.

## 2. Materials and Methods

We conducted this review in accordance with the PRISMA 2020 guidelines and were informed by the protocols from established systematic review reporting frameworks, as outlined by Page et al. [35].

### 2.1. Search Strategy

Five databases (Ovid Medline, PubMed, Web of Science, Scopus, and Cochrane) were searched to comprehensively assess published articles [36]. No restrictions were placed on the search period. The search strategy used two approaches. First, we undertook a general search; search terms relating to animal subjects included “camel ^∗^” and “dromedar ^∗^”. For diseases, the terms “zoono ^∗^”, “transboundary”, “emerging”, and “re‐emerging” were initiated using the Boolean operator “OR” with combinations relating to TADs of camels. Disease and population searches were then combined using the Boolean operator “AND” ("Emerging diseases” OR “Re‐emerging diseases” OR “Transboundary diseases” AND “Zoonotic diseases” OR Zoonos ^∗^ AND Camel ^∗^ OR Dromedar ^∗^ AND “Camel‐borne diseases” AND “Public health impact” OR “Animal‐to‐human transmission” OR “One Health approach” AND “Prevention and control strategies” OR “Camel trade” OR “Disease outbreaks in camels"). This was developed to capture general literature on zoonotic TADs of camels.

Second, disease‐specific searches were conducted for TADs listed by FAO and WOAH with known zoonotic potential and significant public health impact, such as MERS, RVF, and HEV. Search strings for specific diseases were generated by combining the scientific name(s) of the disease (such as MERS), alternative name or known abbreviation, if any (e.g., MERS), and aetiology (e.g., MERS‐CoV), and then combined with the study population (Camel ^∗^ OR Dromedar ^∗^) using the Boolean operator “AND”. We then assessed the inclusion criteria and search phrases before proceeding with the searches.

### 2.2. Inclusion and Exclusion Criteria

Prevalence and/or risk‐factor studies, which may be a case study, longitudinal study, or outbreaks with natural infection of a zoonotic TAD detected in camels transported across country borders, were considered satisfactory for the review. The exclusion criteria included diseases not considered in the FAO, WOAH list of TADs, articles not written in English, review papers, conference proceedings, and grey literature such as media articles and unpublished government reports.

### 2.3. Study Selection

**Figure 1:**
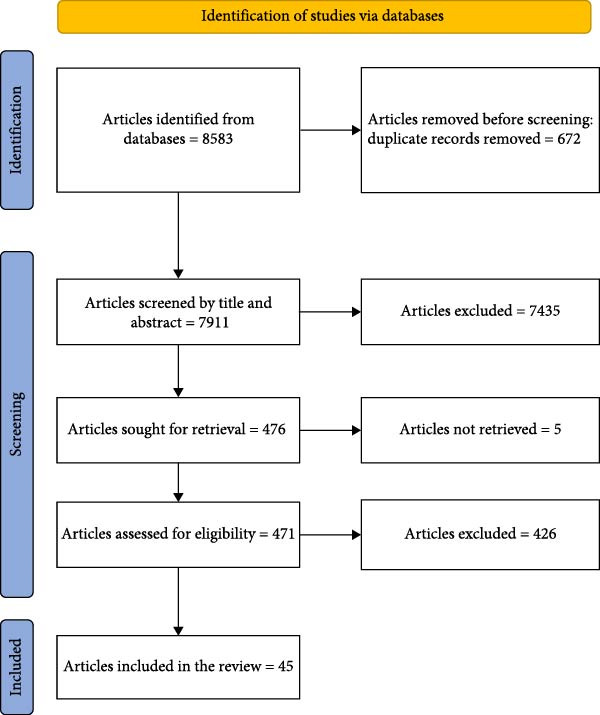
Preferred reporting items for systematic reviews and meta‐analyses (PRISMA) flow diagram detailing the number of articles on camel TADs retrieved and selected for data extraction.

All articles, irrespective of language barriers and timeframe, were included. After searching, retrieved articles were exported to Rayyan software for systematic review [37]. The records were merged into a single library, and duplicates were checked. A second de‐duplication was also carried out using Rayyan. Articles’ titles and abstracts were separately reviewed by two reviewers, who then verified if the work satisfied the inclusion criteria. The study selection process is illustrated in Figure 1, which presents the PRISMA flow diagram detailing the number of articles on TADs retrieved and included for data extraction.

### 2.4. Quality Assessment

A full‐text review was conducted to evaluate the quality of the eligible studies. The Joanna Briggs Institute (JBI) Critical Appraisal Checklist was adopted to assess prevalence studies [38]. The quality assessment of the included studies was conducted independently by two researchers. The evaluation results were then combined by one author using a Microsoft Excel spreadsheet. Ten modified questions from the JBI critical appraisal tool, with the options “yes”, “no”, “unclear”, and “not applicable”, were incorporated to guide us during the assessment. The proportions of yes responses among all yes, no, and unclear responses for each article were used to create three groups based on a scoring range: low (<50%), medium (≥50% to ≤70%), and high (> 70%). Disagreements were resolved through discussion.

### 2.5. Data Extraction and Statistical Analysis

From each included study, the following variables were extracted: author and year of publication, study period, geographical location, the specific TAD investigated, the reported prevalence of the TAD, origin of camel samples, age, sex, the type of biological samples or specimen analysed (serum, nasal swab, rectal swab, milk, faeces or urine), camel management system (imported or mixed with local) and diagnostic method or laboratory technique used for pathogen detection (serology or molecular).

We calculated the prevalence of TADs as proportions and 95% confidence intervals (CIs). As we anticipated considerable between‐study heterogeneity [39], a random‐effects model with logit transformation was used to estimate pooled prevalence effect sizes. CIs around the pooled prevalence estimates were calculated using the normal approximation method based on the summary measure (Supporting Informations 1–4: Figures S1–S4). Heterogeneity was assessed using between‐study variance (*τ* ^2^) and the proportion of total variance due to heterogeneity (*I* ^2^ statistic). Publication bias was assessed using funnel plots (Supporting Information 5: Figure S5). To explore potential sources of between‐study heterogeneity, we conducted meta‐regression analysis. Univariate and multivariable meta‐regression models evaluated the influence of study covariates, including diagnostic technique (serological vs. molecular), type of TADs, and camel management (e.g., imported vs. local or mixed) (Tables 1 and 2). All statistical analyses were conducted in R version 4.3.1 using the functions for meta‐analysis of proportions.

**Table 1:** Univariate meta‐regression analysis for TADs prevalence estimate.

Covariate	Estimate	SE	I^2^ (%)	*R* ^2^ (%)	*p*‐Value
Year	−0.02	−0.23	99.69	0	0.798
Study region	—	—	99.69	0	0.924
Algeria	1.94	−7.39	—	—	0.446
Nigeria	0.82	−5.76	—	—	0.576
TAD type	—	—	99.73	0	0.508
MERS	2.37	−4.93	—	—	0.059
RVF	2.06	−5.72	—	—	0.158
Management	—	—	99.7	0	0.997
Imported	0.14	−7.20	—	—	0.938
Imported/local	0.13	−7.24	—	—	0.943
Lab technique	—	—	99.63	12.11	0.021
Molecular	−0.34	−9.61	—	—	0.889
Serology	1.58	−9.50	—	—	0.516

**Table 2:** Multivariable meta‐regression analysis for TADs prevalence estimate.

Covariate	Estimate	SE	I^2^ (%)	*R* ^2^ (%)	*p*‐Value
Overall model	—	—	99.5	36.38	0.0002
TAD type	—	—	—	—	—
MERS	4.10	1.06	—	—	0.0001
RVF	2.27	1.23	—	—	0.066
Management	—	—	—	—	—
Imported	−3.21	1.85	—	—	0.083
Imported/local	−2.53	1.82	—	—	0.164
Lab technique	—	—	—	—	—
Molecular	−4.72	2.45	—	—	0.054
Serology	−1.24	2.33	—	—	0.594

## 3. Results

### 3.1. Search Results

The search returned a total of 8583 publications from five databases. The titles and abstracts of 7911 papers were screened to ensure compliance with the target population after removing 672 duplicates. Subsequently, 7453 papers were excluded for neither involving camels (imported or engaged in cross‐border movement) nor the camel source/rearing system and TAD criteria. A total of 471 papers were then assessed for eligibility by the reviewers, of which 45 were subjected to a data extraction process based on the inclusion and exclusion criteria (Figure 1). Overall, Table 3 summarise the key findings by disease type and region, while the full dataset is presented in Supporting Information 6: Table S1.

**Table 3:** Summary of studies on transboundary‐related TADs detected in camels.

Author/year	Study period and location	TADs	Camel source/raring system	Samples /specimen	Method/ technique
Viral TADs					
[40]	Tunisia 2016	Bluetongue and West Nile fever	Pastoralists intercepted in Tunisian cities bordering Libya and Algeria	Serum	ELISA
[41]	Egypt	Bovine viral diarrhoea, Rift Valley fever	Sudan and Egypt	Serum	ELISA
[42]	Kenya	Camel pox	Kenya/Somali border	Skin lesion	Neutralisation test
[43]	Saudi Arabia, 2017–2019	Hepatitis E	Imported and local farms in Sudan, Djibouti, Saudi Arabia	Serum	RT‐PCR
[44]	1983 1983–1984 1997 1992–2015 2013 2012–2015	Hepatitis E	Sudan, Somalia, Egypt, Kenya, Pakistan, UAE	Serum Faeces	ELISA, RT‐PCR
[45]	SA, 2016–2018	Hepatitis E	Imported and local farms in Sudan, Djibouti, Saudi Arabia	Serum	ELISA
[46]	SA, 2017–2018	Influenza A	Imported and local farms in Sudan, Djibouti, Saudi Arabia	Nasal swabs	RT‐PCR
[28]	SA, 2017–2019	MERS	Imported and local farms in Sudan, Djibouti, and Saudi Arabia.	Serum Faeces	Microneutralization assay and RT‐PCR
[47]	UAE, April–March 2015	MERS	UAE, Oman, Unknown	Nasal swabs	PCR
[48]	Ethiopia. January 2017–September 2020	MERS	Ethiopia border regions	Nasal and turbinate swabs	PCR
[49]	Eastern Africa. June–July 1984, June–July 1997, January 1983–December 1984	MERS	Eastern Africa	Serum	ELISA and microneutralization assay
[50]	Ethiopia, 2015, 2017	MERS	Sudan, Qatar	Serum nasopharyn‐geal nasal	ELISA, RT‐PCR
[51]	Egypt, April 2016–March 2018; Senegal, August–September, 2017; Uganda, February–March 2017; Tunisia, December 2015–January 2018; KSA, November 2015–October 2016; Iraq, January–17	MERS	Egypt/Sudan	Serum and nasal swabs	Microneutralization assay for serum, RT‐PCR for nasal swabs
[52]	Iran, 2014	MERS	Imported, Pakistan‐Iran border	Nasal and rectal swabs	RT‐PCR
[53]	KSA, January 2016–Mar 2018	MERS	Local and imported from Somalia/Sudan	Nasal and rectal swabs	RT‐PCR
[54]	UAE, February–September 2014	MERS	UAE, UAE borders with KSA/Oman	Nasal swabs	RT‐PCR
[55]	Burkina Faso, February–Mar, 2015	MERS	Burkina Faso, Ethiopia, Morocco	Serum and nasal swabs	Microneutralization assay and RT‐PCR
[56]	January 2015–December 2016	MERS	Imported‐Sudan	Serum	ELISA
[57]	Egypt, Jun 2014–February 2016	MERS	Imported/local‐ Somalia/Sudan/Ethiopia	—	—
[58]	KSA, 1993, 2014	MERS	KSA, Australia	Serum	Microneutralization assay Pseudoparticle neutralisation test
[59]	Sudan, Morocco. 2000–2009	PPR	Sudan	Lungs, liver, spleen	RT‐PCR
[60]	Maritania, October–10	RVF	Mauritania	Serum	Nested RT‐PCR
[61]	Maritania, October–December 2010	RVF	Mauritania	Serum	PCR
[62]	Kenya, 2000–2007	RVF	Kenya high trade/border	Serum	ELISA
[33]	Nigeria, November 2016–April 2017	RVF	Nigeria/Sahel region	Serum	ELISA
[63]	Nigeria	RVF	Nigeria/Chad/Niger	Serum	ELISA
[64]	Nigeria	Influenza A	Nigeria/Sahel region	Serum	ELISA
[65]	Nigeria, November 2016–April 2017	Hepatitis E	Nigeria/Sahel region	Serum	ELISA
[34]	Nigeria	MERS	Nigeria/Sahel region	Serum	ELISA
[66]	Iran, January–June 2019	HEV	Iran, imported from Pakistan and Afghanistan	Blood and liver	RT‐PCR
[67]	Algeria, June 2021–August 2022	CCHV	Imported, local and unknown	Serum	ELISA
[68]	Algeria, June 2021–August 2022	RVF	Imported and local	Serum	ELISA
[69]	KSA	Coronaviruses (CoVs)	Imported from Djibouti and Sudan	Nasal swabs	RT‐PCR
[70]	Nigeria	CCHV	Nigeria and neighbouring countries	Serum	ELISA
[71]	Nigeria	MERS	Nigeria, Chad, Libya, Mali, Niger and Sudan	Nasal swabs and serum	–RT‐qPCR –MERSspike pseudoparticle neutralisation test (ppNT)
[72]	Nigeria, October–December 2016	MERS	Niger Republic	Serum	ELISA
Bacterial TADs					
[73]	Egypt, Jun 2018–January 2019	Brucellosis	Imported from Sudan	Serum	Buffered Plate Antigen test, Rose Bengal test, ELISA
[74]	Nigeria	Brucellosis	Herds and slaughterhouses along the Nigeria‐Niger border	Serum	Modified Rose Bengal plate test (RBPT) Serum Agglutination test (SAT)
[29]	Ethiopia	Brucellosis	Ethiopia and from Somalia	Serum	Rose Bengal plate test
[75]	Slovenia 2004	Tuberculosis	Slovenia	—	Tuberculin test
[76]	Eritrea, October–November, 2013 and September–December, 2014	Bovine tuberculosis	Pastoral, herds Eritrean border	—	Tuberculin test
[77]	Algeria. July 2018–Jun 2019	Q‐fever	Algeria/Tunisia border	Serum	ELISA
[78]	Egypt, November 2015 – March 2016	Brucella Q‐fever	Imported from Sudan	Serum	ELISA
Parasitic TADs					
[79]	Egypt, November 2014– February 2016	Toxoplasmosis	Imported	Serum	Latex agglutination test, ELISA
[80]	Kenya, March–92	Trypanosomosis	Imported	Blood	Xenodiagnosis DNA probe

### 3.2. Geographic Distribution of TADs

Overall, 45 articles published between 1994 and 2024 reporting TAD pathogens in camels were included in this study (Figure 2). Most studies were from Egypt, Nigeria, Saudi Arabia, and Kenya (Figure 2A), and the surge in research output began around 2014, notably due to RVF and MERS (Figure 2B). Fifteen prominent TADs were included in the studies; MERS (34%) dominated the literature, followed by RVF (15%), hepatitis E (11%), and brucellosis (9%) (Figure 2C). Overall, the study revealed that 97% of camel trade, importation, and movement occur between the Middle Eastern countries, Northern, Eastern, and parts of Western Africa. Among the 21 countries included in this review, those in the Middle East and North Africa (MENA) region were most prominently represented, with camel exports concentrated primarily in the Sahelian region. The leading exporters identified in the study were Sudan (29.7%), Somalia (16.2%), and Nigeria (13.5%).

Figure 2Study characteristics: (A) geographical distribution of included studies, (B) year‐wise distribution of studies on TADs in camels from 1994 to 2024, and (C) country‐wise distribution of studies on TADs in camels. BT, bluetongue disease; BVD, bovine viral diarrhoea; CCHV, Crimean Congo haemorrhagic fever; CP, camel pox; HEV, hepatitis E virus infection; MERS, Middle East respiratory syndrome; PPR, *peste des petits* ruminant; RVF, Rift Valley fever; TB, tuberculosis; Toxo, toxoplasmosis; Tryps, trypanosomosis; WNF, West Nile fever.

**Figure:**
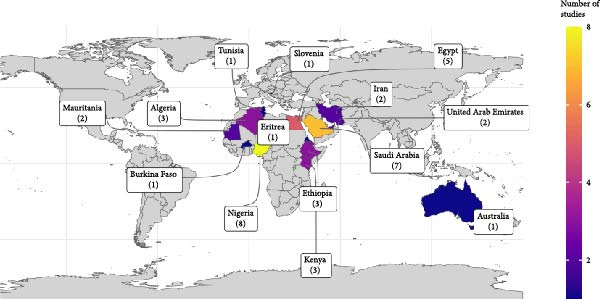
(A)

**Figure:**
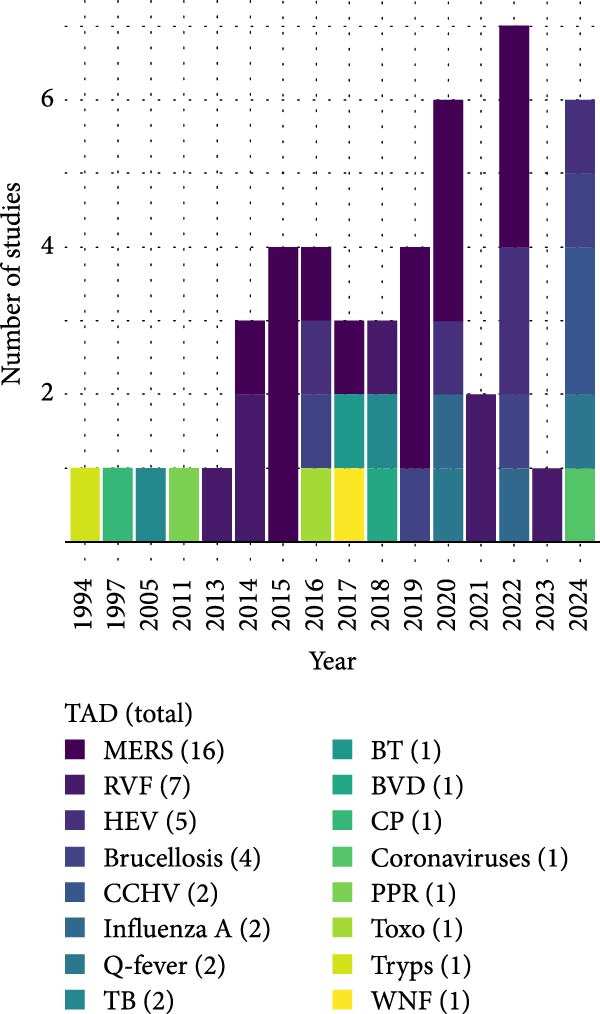
(B)

**Figure:**
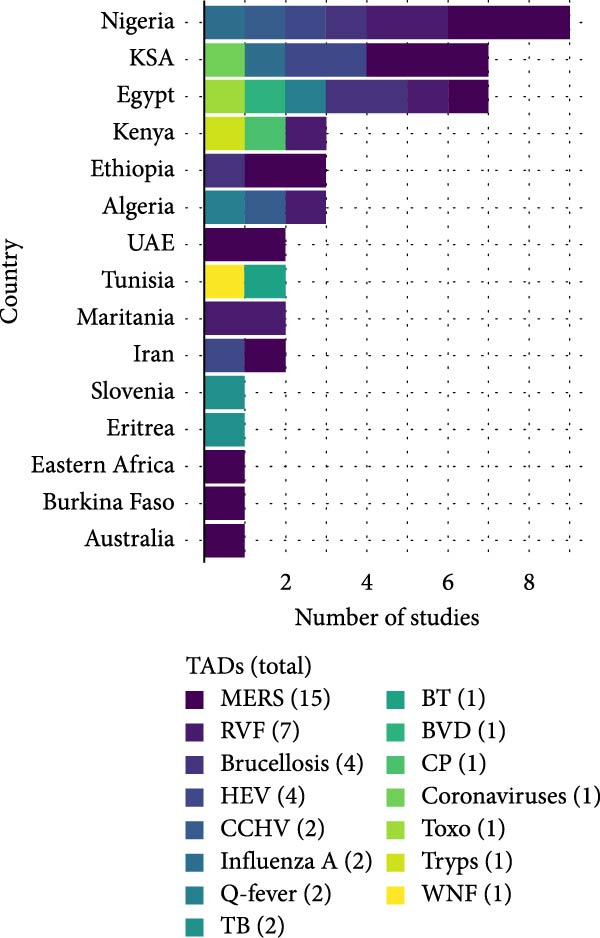
(C)

### 3.3. Distribution of TADs by Age, Sex and Samples Analysed

The biological samples examined for TAD detection across studies were mostly serum (72.7%) and nasal swabs (27%). Other biological samples analysed included skin lesions, faeces, rectal swabs, milk, urine, liver, lungs, and spleen. Sex was reported in 10 (27%) of the included studies, with a greater proportion of male camels (64.4%) sampled compared to females (35.6%). Age was specified in eight studies (21.6%), where camels aged 1 year and above accounted for the majority (94.6%), while only 5.4% were under 1 year.

### 3.4. Distribution of TADs by Disease Type

#### 3.4.1. Viral TADs

Viral TADS were the most reported, comprising 29 articles (78.7%). MERS was the most frequently studied (*n* = 16, 34%), with camels mainly originating from Sudan (42.8%), Saudi Arabia (28.6%), and the Horn of Africa (Somalian and Ethiopian borders, 21.4%). Nasal swabs and serum (64.3% each) or a combination (42.8%) were the most tested specimens analysed for laboratory detection of MERS. Faecal, rectal, milk, and urine samples have also been screened for MERS [57]. Reverse transcription polymerase chain reaction (RT‐PCR) was used in 71.4% of studies for viral detection, typically targeting the E gene and confirmed by the open reading frame (ORF) 1a or N gene. Serological detection relied on microneutralization assay (MNA) (42.9%) and enzyme‐linked immunosorbent Assay (ELISA) (28.6%), often in combination (42.9%). Overall, reported MERS seroprevalence ranged from 71% in Egypt [57] to 100% in Nigeria [34], with higher rates in older camels (96.5%) than younger ones and in females (94%) compared to males (91%) [28]. However, RT‐PCR positivity was more common in males (24.3%) [28] in some studies, though findings were inconsistent across sex and age [47].

We identified 7 (15%) studies on RVF, with serum used exclusively. ELISA was more commonly employed than PCR. Seroprevalence ranged from 7.1% in Mauritania [60] to 94.8% among camels from Sahelian countries [68]. The seroprevalence of RVF was not consistent with the sex or age of camels. El Bahgy et al. [41] reported higher seroprevalence in younger camels (19.2%) than in adults (12.6%) and a higher seroprevalence in males (21.4%) than in females (9.2%), while Musa et al. [63] detected a lower seroprevalence in younger camels (6.9%) compared to adults (21.1%) and a lower seroprevalence in males (20%) than in females (9.2%). Molecular analysis has detected RVF virus strains in studies conducted in West Africa in Zimbabwe, Kenya, South Africa, and Uganda [60],[61]].

There were five articles (10.6%) on the hepatitis E virus (HEV), which was detected in camels imported from various parts of Africa (Sudan, Somalia, Egypt, and Kenya) and the Middle East (Saudi Arabia, the UAE, and Pakistan). Sarani et al. [66] detected the highest seroprevalence (56.6%) from serum samples using ELISA. Rasche et al. [44] also detected HEV in 1.9% of faecal samples from the UAE using RT‐PCR in another study. Sex as a risk factor for HEV in camels was inconsistent among studies. Adamu et al. [64] reported higher seroprevalence in females than males (45.8% vs. 25%). However, in the study by ElKafrawy et al. [45], sex was significantly associated with the detection of HEV (*p*‐value = 0.015, OR = 6.7), where males (31.6%) appeared more infected than females (13.4%). Although older camels (> 3 years) showed a higher seroprevalence of HEV [45, 64], the age of the camels was not significantly different.

Two studies (5.4%) on influenza A in camels were identified. Alghamdi et al. [46] detected influenza A virus in 1.7% of nasal swab samples collected from camels imported from Sudan and Djibouti and local farms in Saudi Arabia using RT‐PCR. Following partial genome sequencing, isolates showed a close relationship between human and swine influenza A isolates from different countries. Another study [64] carried out on camels across the Nigeria‐Sahel border region revealed 10.3% seroprevalence from serum analysed. Single studies also reported bluetongue (25.8%) and WNF (5.9%) in Tunisian camels [40], bovine viral diarrhoea (BVD) (33%) in Egyptian camels [41], camelpox (15.5%) in Kenyan camels [42], and PPR (77.6%) in Sudanese camels [59].

#### 3.4.2. Bacterial TADs

A total of three (8.1%) studies focused on brucellosis, with seroprevalence ranging from 4.9% in camels from Ethiopia/Somalia [29], 8.6% in camels imported from Sudan [73], and 11.5% in the Nigeria‐Sahel border [74]. Two studies (5.4%) assessed tuberculosis, detecting positive skin test responses in Slovenia [75] and Eritrea [76], indicating possible cross‐border transmission. Q‐fever was reported in two studies (2.7%), with 75% seroprevalence at the Algeria‐Tunisian border and 4.3% in Egypt [77] and [78]. Male and older camels tended to have higher seroprevalence.

#### 3.4.3. Parasitic TADs

Two studies reported parasitic infections: toxoplasmosis [81] and trypanosomosis [82]. Toxoplasmosis had a 35.7% seroprevalence in imported camels [79], while *Trypanosoma simiae* was detected at a seroprevalence of 14.3% in a small sample size using xenodiagnosis and a DNA probe [80].

### 3.5. Pooled Prevalence Estimates

**Figure 4:**
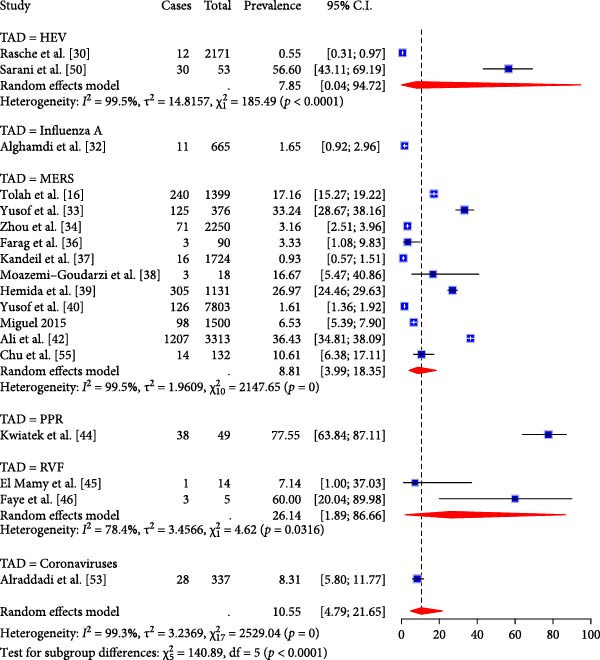
Subgroup analysis of TADs by molecular detection.

The seroprevalence was higher in studies that adopted serological assays for screening for TADs (44.73%, 95% CI: 27.24; 63.63) than the prevalence observed using molecular techniques (10.55%, 95% CI: 4.79; 21.65) in the subgroup analysis (Figure 3A). Regarding the potential variations among the different TADs considered in this study, MERS seropositivity had the highest pooled effect size (84.12%, 95% CI: 67.72; 93.04, I^2^ = 99.1%, *τ* ^2^ = 2.3, *p* < 0.0001) while RVF and HEV were 37.88% (95% CI: 10.10; 76.80, I^2^ = 98.3%, *τ* ^2^ = 3.7, *p* < 0.0001) and 16.02% (95% CI: 3.56; 49.66, I^2^ = 98.7%, *τ* ^2^ = 2.8, *p* < 0.0001) respectively (Figure 3B). Subgroup analysis of TAD prevalence based on molecular detection revealed only five diseases, with studies on RVF showing the highest pooled effect size (26.14%, 95% CI; 1.89; 86.66, I^2^ = 78.4%, *τ* ^2^ = 3.5, *p* = 0.03) compared to MERS (8.81, 95% CI; 3.99; 18.35, I^2^ = 99.5%, *τ* ^2^ = 1.96, *p* = 0) and HEV (7.85%, 95% CI; 0.04; 94.72, I^2^ = 99.5%, *τ* ^2^ = 14.8, *p* < 0.0001) even though studies on MERS were the most predominant (Figure 4). The high I^2^ values (> 90%) across pooled estimates suggest substantial heterogeneity, likely due to differences in diagnostic methods, camel management systems, and study designs. Moreover, sensitivity analyses stratified by diagnostic technique (serology vs. molecular) revealed significant variation in prevalence estimates (Figure 3A).

Figure 3(A) Subgroup analysis of the overall TADs study by laboratory technique. (B) Subgroup analysis of TADs studies by serology.

**Figure:**
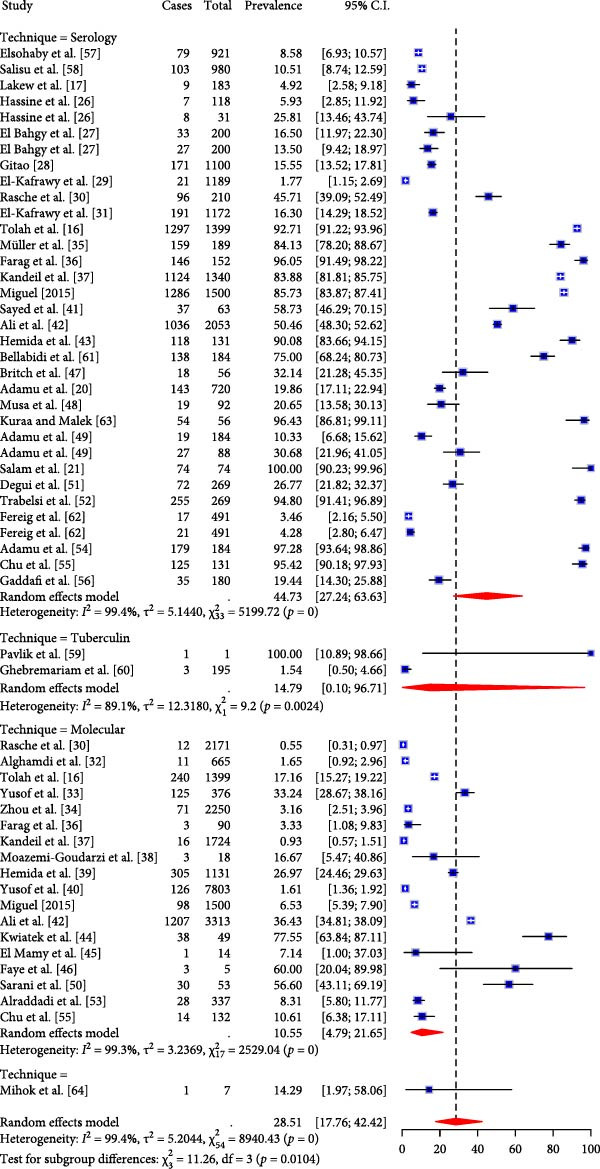
(A)

**Figure:**
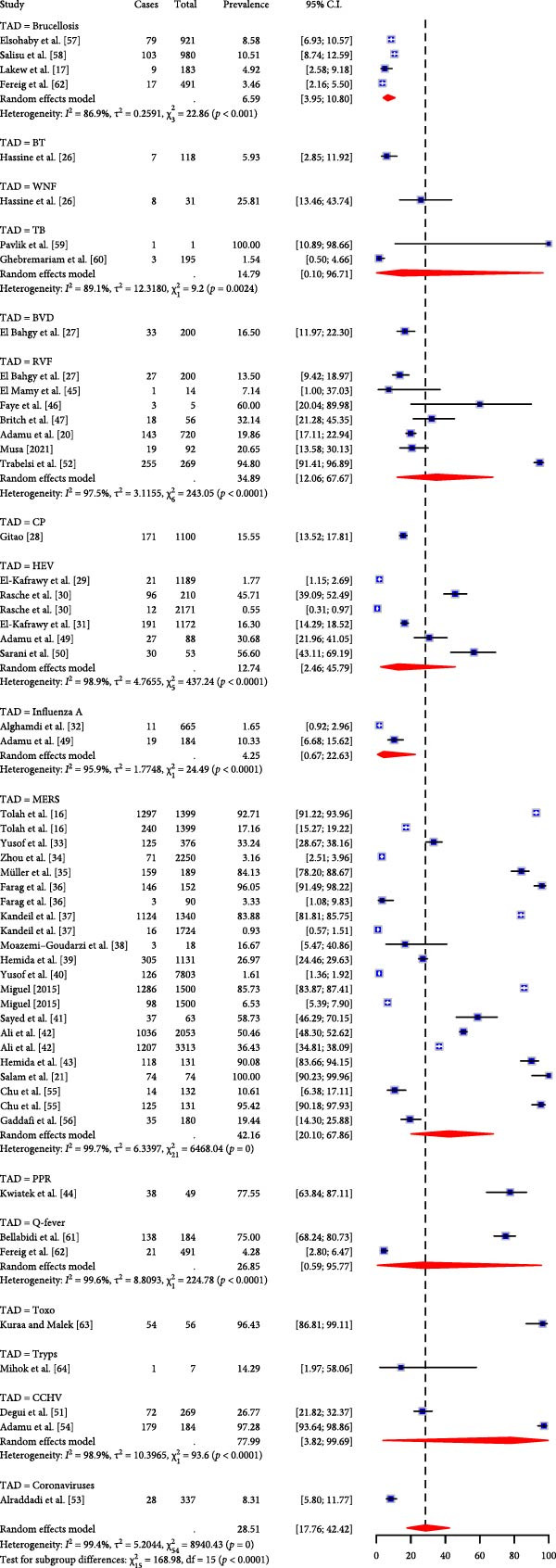
(B)

### 3.6. Meta‐Regression

Univariate meta‐regression analyses (Table 1) revealed that study period (*p* = 0.798), geographical region of study (*p* = 0.924), type of TAD (*p* = 0.508), and camel management system (*p* = 0.997) were not significantly associated with pooled prevalence estimates and explained none of the observed heterogeneity (*R* ^2^ = 0.00%). Diagnostic technique was the only covariate significantly associated with prevalence estimates (*p* = 0.02), accounting for 12.11% of the heterogeneity observed (*p* = 0.02, *R* ^2^ = 12.11%). This finding indicates that serological methods tend to yield higher prevalence rates than molecular techniques and underscores the need for harmonised diagnostic protocols in camel surveillance. A multivariable mixed‐effects meta‐regression model incorporating diagnostic and laboratory techniques, the type of TAD, and the camel management system explained a larger proportion of heterogeneity. All three covariates were significantly associated with the prevalence of TADs and accounted for 36.38% of the heterogeneity (*R* ^2^ = 36.38%, *p* < 0.001) (Table 2).

## 4. Discussion

### 4.1. Key Findings

This meta‐analysis and systematic review identified 15 TADs related to cross‐border camel movement, with viral pathogens predominating in the literature. MERS‐CoV was the most reported and widespread infection, especially in camels in the Horn of Africa and the Middle East, indicating both its endemic circulation and the extent of camel trade between these regions and others. RVF virus and HEV were also regularly identified in several countries, and it is important to note that camels were identified as potential reservoirs or bridging hosts of emerging zoonoses of public health concern. Aggregated seroprevalence rates were considerably higher than molecular diagnostic prevalence rates, and the diagnostic method was the most significant source of heterogeneity across studies, highlighting the impact of surveillance and diagnostic methods on reported disease burden.

Global camel populations have grown significantly over the past three decades, driven by increasing demand for camels and camel products, particularly in the Middle East and Africa [10, 83, 84]. The Horn of Africa, including Ethiopia, Eritrea, Somalia, Djibouti, Kenya, Sudan, South Sudan, and Uganda, remains the primary source of camel exports and hosts the world’s largest camel population [85]. Despite this, research output from the region remains limited, suggesting potential under‐investment in disease surveillance relative to the scale of camel production and trade.

Studies yielded inconsistent outcomes due to varying designs, making direct comparisons between studies challenging. While some studies reported higher seroprevalence of TADs in male camels than in females [64, 77], these outcomes may have been influenced by several factors. For instance, female camels are kept primarily for breeding and milk production, making them the predominant sex encountered during sampling. In contrast, male camels are primarily used for meat production and transportation; hence, they are the predominant sex found in slaughterhouses, quarantine facilities, and cross‐border trade. In terms of age, calves are more susceptible to infection than older camels due to their immature immune systems and may therefore shed more TAD pathogens [86]. Moreover, older camels exhibited long‐lasting immune responses against diseases from multiple reinfections [83].

Since it was first identified in Saudi Arabia in 2012, [87] MERS‐CoV has remained a major transboundary disease associated with cross‐border camel trade and has caused at least 947 human fatalities globally [88]. Bats are believed to be the original reservoir of Middle East respiratory syndrome coronavirus (MERS‐CoV), but the virus now primarily circulates in dromedary camels, which serve as the main source of human infections ([89, 90]). This review identified studies reporting high MERS‐CoV seroprevalence in camels, mainly in Africa and the Middle East, suggesting the endemicity of the virus across the region. This may be attributed to the robust international camel trade between Middle Eastern countries and those in the Horn of Africa [32]. Pastoralists in the GHA control 60% of the world’s dromedary camels; Somalia alone is home to one‐third of the world’s dromedary camels and a major hub for cross‐border movement and export [91]. Furthermore, despite rapid advances in MERS‐CoV detection techniques such as serological assays and RT‐PCR, research on camel TADs remains substantially limited. This may be due to security challenges in accessing the region and the limited availability of serological and molecular diagnostic facilities and capacity.

Rift Valley fever virus (RVFV) and hepatitis E virus (HEV) are significant emerging zoonotic pathogens of global public health significance and major causes of morbidity, particularly in high‐risk groups, including farmers, herders, veterinarians, and butchers [23, 92]. The detection of these pathogens in camels transported across the Middle East, Africa, and the Sahelian region [43, 64] indicates that camels are a potential source for transmission to humans, and the movement of camels may be a key factor in the dissemination of these viruses.

Similarly, although limited information is available on the circulation of the influenza A virus in camels, evidence supports the virus’s capacity to infect these animals. Whilst only two studies [33, 46]; have reported detection of the virus in nasal swabs and serum, the ecology of this TAD in camels remains poorly understood, underscoring the need for enhanced proactive surveillance. BT, WNF, BVD, and PPR were among the other viral TADs reported in transported camels. These infectious diseases primarily affect cattle, sheep, and goats. However, the detection of these viruses in imported camels warrants further investigation to determine their role in viral circulation and potential transmission pathways to livestock and humans.

The most prominent bacterial disease reported in this review was brucellosis, which is not only zoonotic but also severely affects reproduction in animals, resulting in stillbirths and reduced milk production, impacting farmers and livestock owners worldwide [93]. Although camels may be considered secondary hosts of *Brucella* spp. [94, 95], they can transmit the disease to humans and other animals. The major regions where infected camels originated were Sudan, Somalia, Ethiopia, and the Nigeria‐Sahel border [29, 73, 74].

Camels moved by pastoralists in Tunisian cities bordering Algeria and Libya tested positive for bovine tuberculosis [40], likely due to prolonged contact with other animals. The 75% seroprevalence of Q‐fever (a neglected zoonotic bacterial TAD) in imported camels demonstrates that the disease can spread through cross‐border camel movement [77]. Although exposure to *C. burnetii* is common among humans and livestock; it remains grossly understudied and rarely reported.

### 4.2. Limitations

Despite efforts to conduct comprehensive and repeatable searches of the published literature, the exclusion of grey literature and non‐English publications may have led to under‐representation of certain regions and disease reports. Additionally, the frequent updates of TAD classifications by relevant authorities may have led us to exclude other TAD articles identified in our initial searches. While serology was widely used across most studies, seropositivity may not accurately reflect the pathogen’s transmissibility or current infection status. Seasonal variations in camel movement, which may influence disease transmission dynamics, were not accounted for in this review. Nevertheless, the data from this review provide a foundation for future studies that aim to assess risk or mitigate the effects of camel‐associated TADs on human and livestock populations.

## 5. Conclusions

TADs are increasing in frequency, and camels represent a significant potential source of cross‐border disease transmission. Despite the Horn of Africa and the Sahelian region being home to ~60% of the global camel population, high‐quality, comprehensive research on the occurrence and prevalence of camel‐associated TADs in these regions remains scarce. The risk of zoonotic TAD transmission is heightened by active camel trade and importation, particularly between the Middle East and Africa, as well as transhumance practices among pastoralists throughout arid and semiarid regions of Africa. These findings underscore the urgent need for improved disease surveillance, risk assessment, and targeted control strategies within camel trade corridors.

### 5.1. Implications and Recommendations

This variation in size, trade intensity, and research activity suggests a lack of investment in surveillance and disease monitoring in key areas of global camel movement. Inadequate information limits the ability to accurately evaluate disease risk and can slow the identification of new or recurring zoonoses. We recommend regional harmonisation of camel disease surveillance protocols, investment in molecular diagnostic capacity, and formalisation of camel trade routes to reduce informal movement and associated disease risks. Ultimately, there is a need to increase research on TADs, with emphasis on longitudinal studies, genomic surveillance, and socioeconomic impact assessments that incorporate a collaborative One Health approach aimed at improving understanding and developing targeted control strategies.

## Ethics Statement

This study is a systematic review based on analysis of previously published data and did not involve animals directly.

## Conflicts of Interest

The authors declare no conflicts of interest.

## Author Contributions

**Alex A. Adikwu**: conceptualisation, formal analysis, investigation, methodology, validation, visualisation, writing – original draft, writing – review and editing. **Theophilus I. Emeto**: supervision, validation, writing – review and editing. **Paul F. Horwood**: supervision, validation, writing – review and editing. **Andrew M. Adamu**: conceptualisation, methodology, writing – review and editing. **Olajide A. Owolodun**: conceptualisation, investigation, validation, supervision. **Oyelola A. Adegboye**: conceptualisation, supervision, formal analysis, methodology, writing – review and editing.

## Funding

No funding was received for this manuscript.

## Supporting Information

Additional supporting information can be found online in the Supporting Information section.

## Supporting information


**Supporting Information 1** Figure S1: Forest plot for the subgroup analysis of TADs by management practice.

**Supporting Information 2** Figure S2: Forest plot for the subgroup analysis of TADs by location.

**Supporting Information 3** Figure S3: Forest plot for the subgroup analysis of TADs by research period.

**Supporting Information 4** Figure S4: Forest plot for the subgroup analysis of TADs by pathogen.

**Supporting Information 5** Figure S5: Funnel plot indicating publication bias in the overall prevalence of TADs.

**Supporting Information 6** Table S1: Summary of studies on transboundary‐related TADs detected in camels.

## Data Availability

The data that support the findings of this study are available from the corresponding author upon reasonable request.
