# Supplementary figures and images for: Transboundary Animal Diseases Associated With Cross‐Border Camel Movement. A Systematic Review and Meta‐Analysis
Source: Transbound Emerg Dis. 2026 Feb 27;2026:6650796. doi: 10.1155/tbed/6650796 (PMC12947666; doi:10.1155/tbed/6650796)

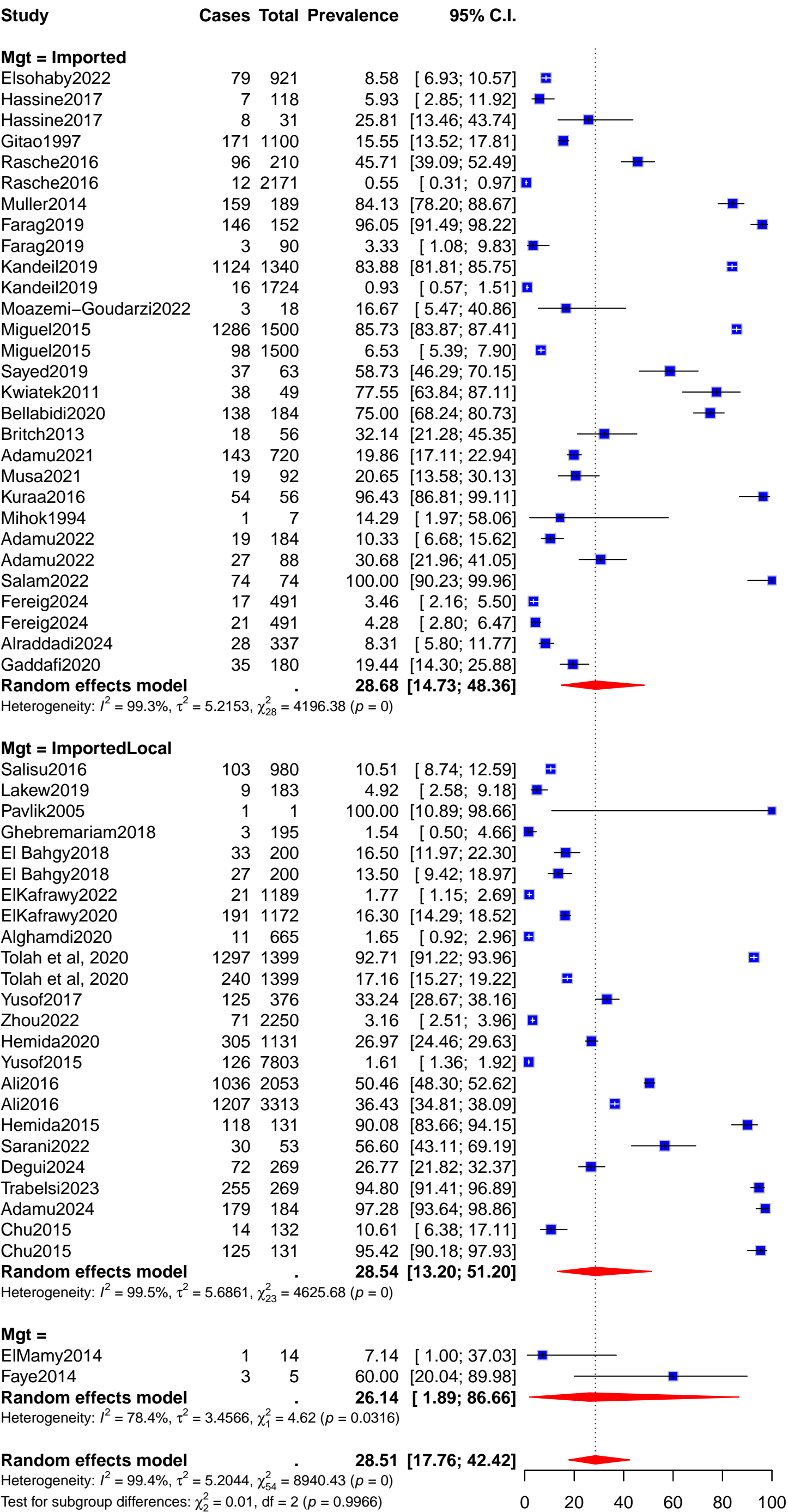

Supplement: Supplementary file 1 — Supporting Information 1 Figure S1: Forest plot for the subgroup analysis of TADs by management practice. [file TBED-2026-6650796-s001.pdf]

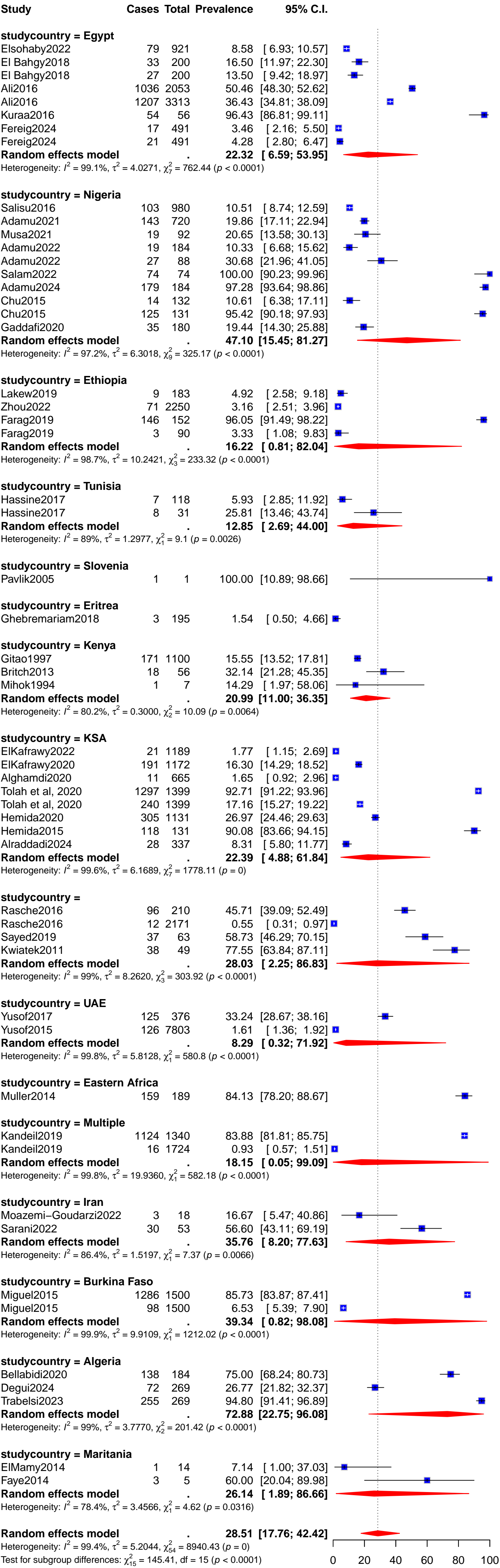

Supplement: Supplementary file 2 — Supporting Information 2 Figure S2: Forest plot for the subgroup analysis of TADs by location. [file TBED-2026-6650796-s002.pdf]

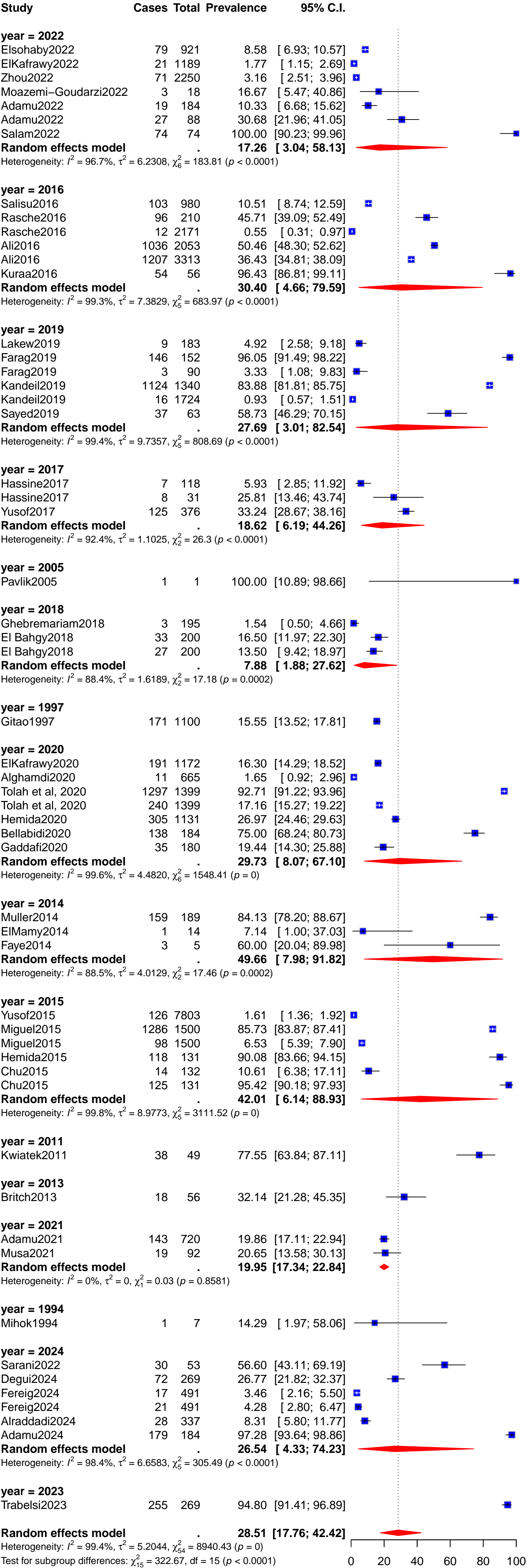

Supplement: Supplementary file 3 — Supporting Information 3 Figure S3: Forest plot for the subgroup analysis of TADs by research period. [file TBED-2026-6650796-s003.pdf]

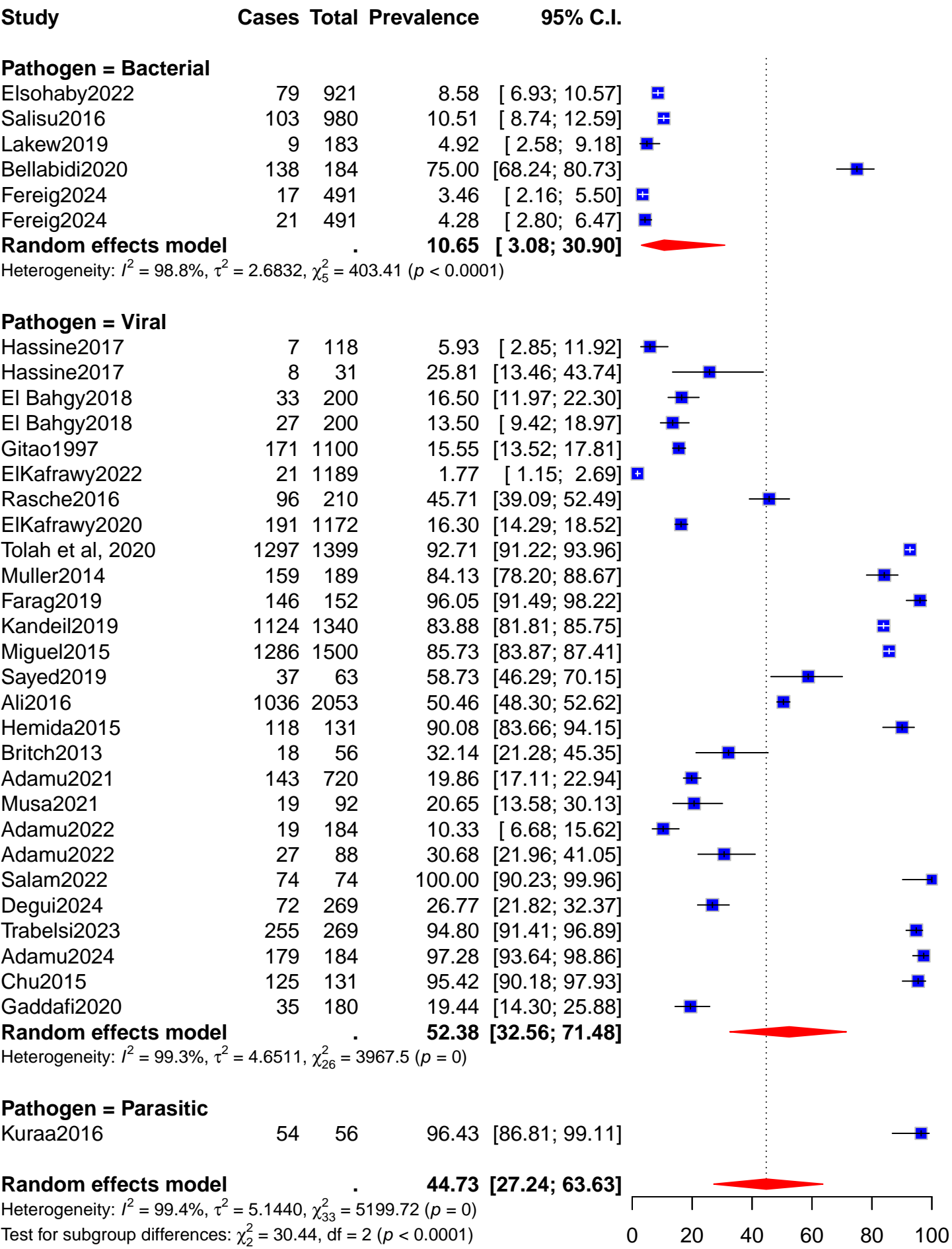

Supplement: Supplementary file 4 — Supporting Information 4 Figure S4: Forest plot for the subgroup analysis of TADs by pathogen. [file TBED-2026-6650796-s004.pdf]

Standard Error

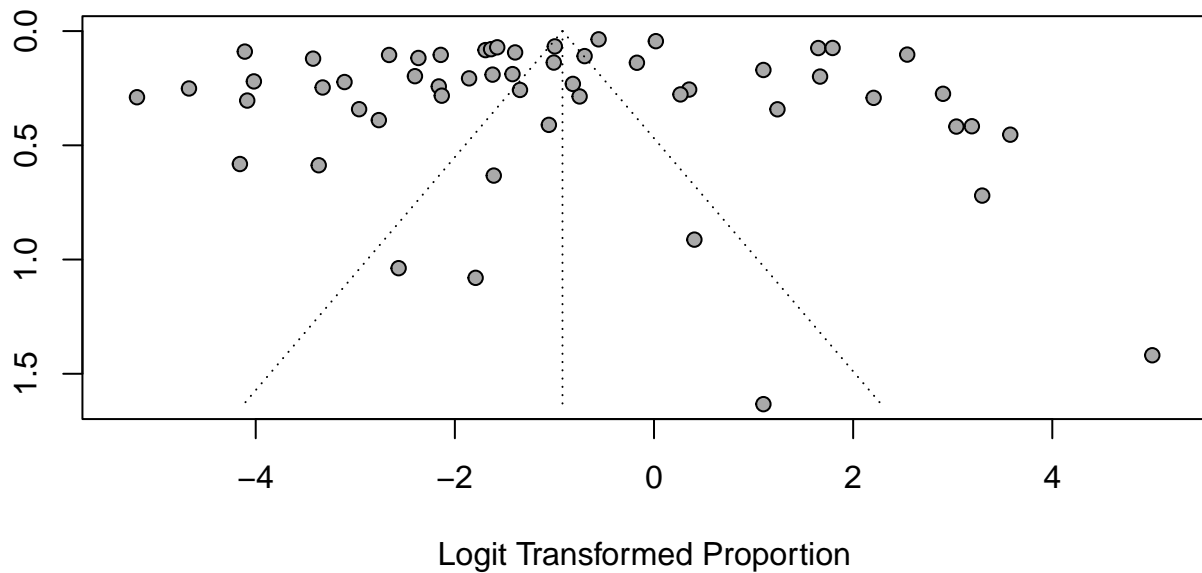

Supplement: Supplementary file 5 — Supporting Information 5 Figure S5: Funnel plot indicating publication bias in the overall prevalence of TADs. [file TBED-2026-6650796-s005.pdf]
