# Supplementary material for: Transboundary Animal Diseases Associated With Cross‐Border Camel Movement. A Systematic Review and Meta‐Analysis
Source: Transbound Emerg Dis. 2026 Feb 27;2026:6650796. doi: 10.1155/tbed/6650796 (PMC12947666; doi:10.1155/tbed/6650796)
Supplement: Supplementary file 6 — Supporting Information 6 Table S1: Summary of studies on transboundary‐related TADs detected in camels. [file TBED-2026-6650796-s006.docx]

**Supplementary list**

Table S1: Summary of studies on transboundary-related TADs detected in camels.

| Author/Year | Study period and Location | TADs | Camel source/Raring system | Samples /Specimen | Sex | Age  (Years) | Method/ Technique | Prevalence (%) |
| --- | --- | --- | --- | --- | --- | --- | --- | --- |
| Elsohaby et al., 2022 | Egypt. Jun 2018 - Jan 2019 | Brucellosis (Bacterial) | Imported from Sudan | Serum | F:M,  510:  411 | 1 - 7 | Buffered Plate Antigen test, Rose Bengal test, ELISA | 8.6 (79/921) |
| Salisu et al., 2016 | Nigeria | Brucellosis (Bacterial) | Herds and slaughterhouses along the Nigeria-Niger border | Serum | NA | NA | Modified Rose Bengal plate test (RBPT)  Serum Agglutination test (SAT) | Herds = 13.3 (63/472) Abattoirs = 9.3 (7/508)  Herds=13.1 (62/472) Abattoir= 8.1(41/508) |
| Lakew et al., 2019 | Ethiopia | Brucellosis (Bacterial) | Ethiopia and from Somalia | Serum | NA | NA | Rose Bengal plate test | 4.9 (9/183) |
| Hassine et al., 2017 | Tunisia 2016 | Bluetongue and  West Nile fever | Pastoralists intercepted in Tunisian cities bordering Libya and Algeria | Serum |  |  | ELISA | BT = 5.9 (7/118) WNF = 25.8 (8/31) |
| Pavlik et al., 2005 | Slovenia 2004 | Tuberculosis (Bacterial) | Slovenia |  |  |  | Tuberculin test | 1 (1 sample) |
| Ghebremariam et al, 2018 | Eritrea Oct-Nov, 2013 & Sep-Dec, 2014 | Bovine Tuberculosis (Bacterial) | Pastoral,  Herds Eritrean border |  |  |  | Tuberculin test | 1.5 (3/195) |
| El Bahgy et al, 2018 | Egypt | Bovine viral diarrhea,  Rift valley fever (Viral) | Sudan & Egypt | Serum | F:M  130:70 | ≤1 = 26  >1 = 174 | ELISA | BVD = 33 (66/200)  Sudan BVDV = 47.5 (57/120)  Local BVDV = 11.3 (9/80)  RVF = 13.5 (27/200)  Sudan RVFV = (16.7) 20/120  Local RVFV = 8.8(7/80) |
| Gitao 1997 | Kenya | Camel pox (Viral) | Kenya/Somali border | Skin lesion |  |  | Neutralization test | 15.5 (171/1100) |
| El‑Kafrawy et al, 2022 | Saudi Arabia. 2017-2019 | Hepatitis E (Viral) | Imported and local farms in Sudan, Djibouti, Saudi Arabia | Serum | F:M  104: 1085 | <1=8, 1-3=229, 3-5=952 | RT-PCR | Imported = 1 (9/893) Local = 4.1 (12/296) <1 = 12.5 (1/8) 1-3 = 3.9 (9/229) 3-5 = 1.2 (11/952) Total = 1.8 (21/1189) |
| Rasche et al, 2016 | 1983 1983–1984 1997 1992–2015 2013 2012–2015 | Hepatits E (Viral) | Sudan, Somalia, Egypt, Kenya, Pakistan, UAE | Serum Faeces |  |  | RT-PCR | Sudan = 0 (0/60) Somalia = 0.9 (1/105) Egypt = 0 (0/50) Kenya = 0.2 (2/889) UAE = (Serum = 0.2 (1/500), (Faeces = 1.9 (5/267) Pakistan = 1.4 (8/567) Total = 0.5 (12/2171 |
|  |  |  |  |  |  |  | ELISA | Sudan = 42.9 (15/35) Somalia = 40 (14/35) Egypt = 62.9 (22/35) Kenya = 31.4 (11/35) UAE = 37.1 (13/35) Pakistan = 60 (21/35) Total = 45.7 (96/210) |
| El‑Kafrawy et al, 2020 | SA. 2016 -2018 | Hepatitis E (Viral) | Imported and local farms in Sudan, Djibouti, Saudi Arabia | Serum |  |  | ELISA | Imported = 22.4 (119/888) Local = 25.4 (72/284) |
| Alghamdi et al, 2020 | SA. 2017 -2018 | Influenza A (Viral) | Imported and local farms in Sudan, Djibouti, Saudi Arabia | Nasal swabs |  |  | RT-PCR | Imported = 2.1 (11/520) Local = 0 (0/145)  Total = 1.7 (11/665) |
| Tolah et al, 2020 | SA. 2017-2019 | MERS (Viral) | Imported and local farms in Sudan, Djibouti, and Saudi Arabia. | Serum Faeces | F:M 364:800 | <1=43 1-2=408  >2=713 | Microneutralization assay | Imported = 86.3 (1085/1257) Local = 7.6 (212/242) F = 94 (342/364) M = 91 (728/800) <1 = 67.4 (29/43) 1-2 = 86.5 (353/408) >2 = 96.5 (688/713)  Total = 92.7 (1297/1399) |
|  |  |  |  |  |  |  | RT-PCR | Imported = 13.3 (154/1257) Local = 35.5 (86/242) F = 12.6 (46/364) M = 24.3 (194/800) <1 = 9.3 (4/43) 1-2 = 28.4 (116/408) >2 = 16.8 (120/713) Total = 12.2 (240/1399) |
|  |  |  |  |  |  |  | RT-PCR & MN | Imported = 11.5 (133/1257) Local = 31.4 (76/242) F = 12.1 (44/364) M = 20.6 (165/800) <1 = 9.3 (4/43) 1-2 = 22.3 (91/408) >2 = 16 (114/713) Total = 14.9 (209/1399) |
| Yusof et al, 2017 | UAE. Apr-Mar, 2015 | MERS (Viral) | UAE, Oman, Unknown | Nasal swabs | F:M 107: 269 |  | PCR | UAE = 28.1 (59/210) Oman = 59.4 (63/106)  Unknown = 5 (3/60)  Total = 33.2 (125/376) |
| Zhou et al, 2022 | Ethiopia. Jan, 2017- Sep, 2020 | MERS (Viral) | Ethiopia border regions | Nasal & turbinate swabs |  |  | PCR | Nasal = 3.5 (61/1766) Turbinate = 2.1 (10/484) |
| Muller et al, 2014 | Eastern Africa. Jun - Jul, 1984, Jun - Jul, 1997, Jan 1983- Dec 1984 | MERS (Viral) | Eastern Africa | serum |  |  | ELISA  Microneutralization Assay | Sudan = 86.7 (52/60) Egypt = 81.4 (35/43)  Somalia = 83.7 (72/86)  Total = 84.1 (159/189)  Sudan = 81.7 (49/60) Egypt = 79.1 (34/43)  Somalia = 81.4 (70/86)  Total = 81.0 (153/189) |
| Farag et al, 2019 | Ethiopia. 2015, 2017 | MERS (Viral) | Sudan, Qatar | Serum Nasopharyn-geal Nasal |  |  | ELISA  RT-PCR | Sudan = 95 (57/62) Qatar = 99 (89/90)  Qatar = 3.3 (3/90) |
| Kandeil et al, 2019 | Egypt. Apr, 2016-Mar, 2018; Senegal. Aug-Sep, 2017; Uganda. Feb-Mar, 2017; Tunisia. Dec 2015-Jan 2018; KSA. Nov 2015-Oct 2016; Iraq. Jan-17 | MERS (Viral) | Egypt/Sudan | Serum & Nasal swabs |  |  | Microneutralization assay for serum, RT-PCR for nasal swabs | Tunisia [S = 87.3 (683/782), N = 0.7 (9/1170)] KSA [S = 81.5 (181/222), N = 3.1 (7/224)]  Jordan [S = 81 (246/304), N = 0 (0/304)] Iraq [S = 43.7 (14/32), N=0 (0/26)] |
| MOAZEMİ-GOUDARZI et al, 2022 | Iran. 2014 | MERS (Viral) | Imported, Pakistan-Iran border | Nasal and rectal swabs |  |  | RT-PCR | 16.7 (3/18) |
| Hemida et al, 2020 | KSA. Jan 2016–Mar 2018 | MERS (Viral) | Local & imported from Somalia/Sudan | Nasal & rectal swabs |  |  | RT-PCR | Nasal = 25.5 (288/1131) Rectal = 7.5 (85/1131) Overall = 27 (305/1131) |
| Yusof et al , 2015 | UAE. Feb– Sep 2014 | MERS (Viral) | UAE, UAE borders with KSA/Oman | Nasal swabs |  |  | RT-PCR | KSA = 1.5 (70/4617) Oman border = 1.1 (31/2853) Abattoir = 8.3 (25/303) Pubic = 0 (0/30) Total =1.6 (126/7803) |
| Miguel et al, 2015 | Burkina Faso. Feb-Mar, 2015 | MERS (Viral) | Burkina Faso, Ethiopia, Morocco | Serum & Nasal swabs |  |  | microneutralization assay  RT-PCR | Burkina Faso = 80.2 (421/525)  Ethiopia = 94.9 (600/632)  Morocco = 77.1 (265/343)  Burkina Faso = 5.3 (28/525)  Ethiopia = 10.2 (64/632)  Morocco = 1.8(6/343) |
| Sayed et al, 2019 | Jan 2015–Dec 2016 | MERS (Viral) | Imported- Sudan | Serum |  |  | ELISA | 58.73 (37/63) |
| Ali et al, 2016 | Egypt. Jun 2014–Feb 2016 | MERS (Viral) | Imported/Local- Somalia/Sudan/Ethiopia | Nasal, Rectal, Milk, Urine, Serum | |  | Microneutralization assay,  RT-PCR | Imported = 89.5 (793/886), Local = 41.3 (243/1167)  Local = 61.3 (1015/1655), 11.6 (192/1658) |
| Hemida et al, 2015 | KSA. 1993, 2014 | MERS (Viral) | KSA, Australia | Serum |  |  | Microneutralization assay  Pseudoparticle neutralization test | KSA = 90.1(118/131)  Australia = (0/25) |
| Kwiatek et al, 2011 | Sudan, Morocco. 2000-2009 | PPR (Viral) | Sudan | Lungs, Liver, Spleen |  |  | RT-PCR | Sudan = 77.6 (38/49) Morocco = 0 |
| Bellabidi et al, 2020 | Algeria. Jul 2018–Jun 2019 | Q-fever (Bacterial) | Algeria/Tunisia border | Serum | F:M 174:10 | <1=15  >1=169 | ELISA  RT-PCT | 75 (138/184)  F = 13.6 (128/174)  M = 100 (10/10)  <1 = 20(3/15)  >1 = 79.9(135/169)  0 |
| El Mamy et al, 2014 | Maritania. Oct-10 | RVF (Viral) | Mauritania | Serum |  |  | Nested RT-PCR | 7.1 (1/14) |
| Faye et al, 2014 | Maritania. Oct-Dec 2010 | RVF (Viral) | Mauritania | Serum |  |  | PCR | 60 (3/5) |
| Britch et al, 2013 | Kenya. 2000-2007 | RVF (Viral) | Kenya high trade/border | Serum |  |  | ELISA | 32.1 (18/56) |
| Adamu et al, 2021 | Nigeria. Nov 2016-Apr2017 | RVF (Viral) | Nigeria/Sahel region | Serum |  |  | ELISA | 19.9 (143/720) |
| Musa et al, 2021 | Nigeria | RVF (Viral) | Nigeria/Chad/Niger | Serum | F:M  57:35 | Yg=29  Ad=63 | ELISA | 20.7 (19/92) F = 21.1 (12/57) M = 20(7/35) Young = 6.9 (2/29) Adult = 21.1 (17/63) Chad = 28 (7/25) Niger = 32.1 (9/28) Nigeria = 7-7 (3/39) |
| Kuraa et al, 2016 | Egypt. Nov2014- Feb2016 | Toxoplasmosis (Parasitic) | Imported | Serum | F:M 29:27 |  | Latex agglutination test  ELISA | 35.7 (20/56) F = 24.1 (7/29) M = 48.2 (13/27)  96.4 (54/56) F = 100 (29/29) M = 92.6 (25/27) |
| Mihok et al, 1994 | Kenya. Mar-92 | Trypanosomosis (Parasitic) | Imported | Blood |  |  | Xenodiagnosis DNA probe | 14.3 (1/7) |
| Adamu et al., 2022 | Nigeria | Influenza A (viral) | Nigeria/ Sahel region | Serum | F:M 83:101 | 0-4 =47, ≥5=137 | ELISA | 10.33 (19/184) F = 6.02(05/83) M = 13.86 (14/101)  0-4yrs = 8.51 (4/47)  ≥5yrs = 10.95 (15/137) |
| Adamu et al., 2022 | Nigeria. Nov. 2016 – April 2017 | Hepatitis E (Viral) | Nigeria/ Sahel region | Serum | F:M 24:64 | 0-15= 85  >15=3 | ELISA | 30.7 (27/88) F = 45.8 (11/24) M = 25 (16/64)  0-15yrs = 29.5 (26/85)  ≥5yrs = 33.3 (1/3) |
| Salam et al., 2022 | Nigeria | MERS (Viral) | Nigeria/ Sahel region | Serum |  |  | ELISA | 100 (74/74) |
| Sarani et al., 2023 | Iran, Jan. – Jun, 2019 | HEV (Viral) | Iran, Imported from Pakistan and Afghanistan | Blood, Liver | F:M 22:31 | <2 =12 ≥2 = 41 | RT-PCR | Overall =56.6 (30/53)  Female= 40.9 (9/22)  Male= 67.74 (21/31)  Blood= 38.89 (7/18)  Liver= 65.71 (23/35)  <2years = 100 (12/12)  ≥2years = 43.9 (18/41)  Domestic camels = 5.56 (1/18)  Imports from Pakistan = 73.9 (12/23)  Imports from Afghanistan = 100 (12/12) |
| Degui et al., 2024 | Algeria, Jun. 2021 – Aug. 2022 | CCHV (Viral) | Imported, Local and Unknown | Serum | F:M 95:174 | <5= 29 ≥5=240 | ELISA | Overall 26.7% (72/269)  Female = 94.73 (90/95)  Male = 94.82 (165/174)  <5years = 82.75 (24/29)  ≥5years = 96.25 (231/240)  Imported = 96.98 (193/199)  Local = 85.96 (49/57)  Unknown = 100 (13/13) |
| Trabelsi et al., 2023 | Algeria. Jun. 2021 – Aug. 2022 | RVF (Viral) | Imported and Local | Serum | F:M 95:174 | <5= 29 ≥5=240 | ELISA | Overall 94.79 (255/269)  Female = 21 (20/95)  Male = 29.8 (52/174)  <5years = 4.5 (2/44)  ≥5years = 31.1 (70/225)  Imported = 35.82 (72/201)  Local = 0 (0/68) |
| Fereig et al., 2024 | Egypt. Nov. 2015 – Mar. 2016 | Brucella (Bacterial)  Q-fever (Bacterial) | Imported from Sudan | Serum | All Male | Adult (>2years) | ELISA | Brucella = 3.5 (17/491)  Q-Fever = 4.3 (21/491) |
| Alraddadi et al., 2024 | KSA | Coronaviruses (CoVs) (Viral) | Imported from Djibouti and Sudan | Nasal swabs | F:M 137:200 | 2 = 118 ≥2 = 219 | RT-PCR | Overall = 8.3 (28/337)  Female = 6.57 (9/137)  Male = 9.5 (19/200)  <2years = 3.92 (4/118)  ≥2years = 10.96 (24/219)  Imports from Sudan = 5.78 (13/225)  Imports from Djibouti = 13.39 (15/112) |
| Adamu et al., 2024 | Nigeria | CCHV (Viral) | Nigeria and neighbouring countries | Serum | F:M 146:38 | <5= 5 ≥5=179 | ELISA | Overall = 97 (179/184)  Female = 78 (144/184)  Male = 19 (35/184)  <5years = 1.6 (3/184)  ≥5years = 96 (176/184) |
| Chu et al., 2015 | Nigeria | MERS (Viral) | Nigeria, Chad, Libya,  Mali, Niger and Sudan | Nasal swabs and Serum | NA | Adults | -RT-qPCR  -MERSspike  pseudoparticle neutralisation test (ppNT) | RT-qPCR (Nasal swabs) = 11 (14/132)  ppNT (Serum) = 95 (125/131) |
| Gaddafi et al., 2020 | Nigeria (Oct. – Dec. 2016) | MERS (Viral) | Niger Republic | Serum | F:M 63/1117 | ≤3= 114 >3= 66 | ELISA | Overall = 19.4 (35/180)  Female = 19.04 (12/63)  Male = 19.69 (23/117)  ≤3years = 19.29 (22/114)  >3years = 19.69 (13/66) |


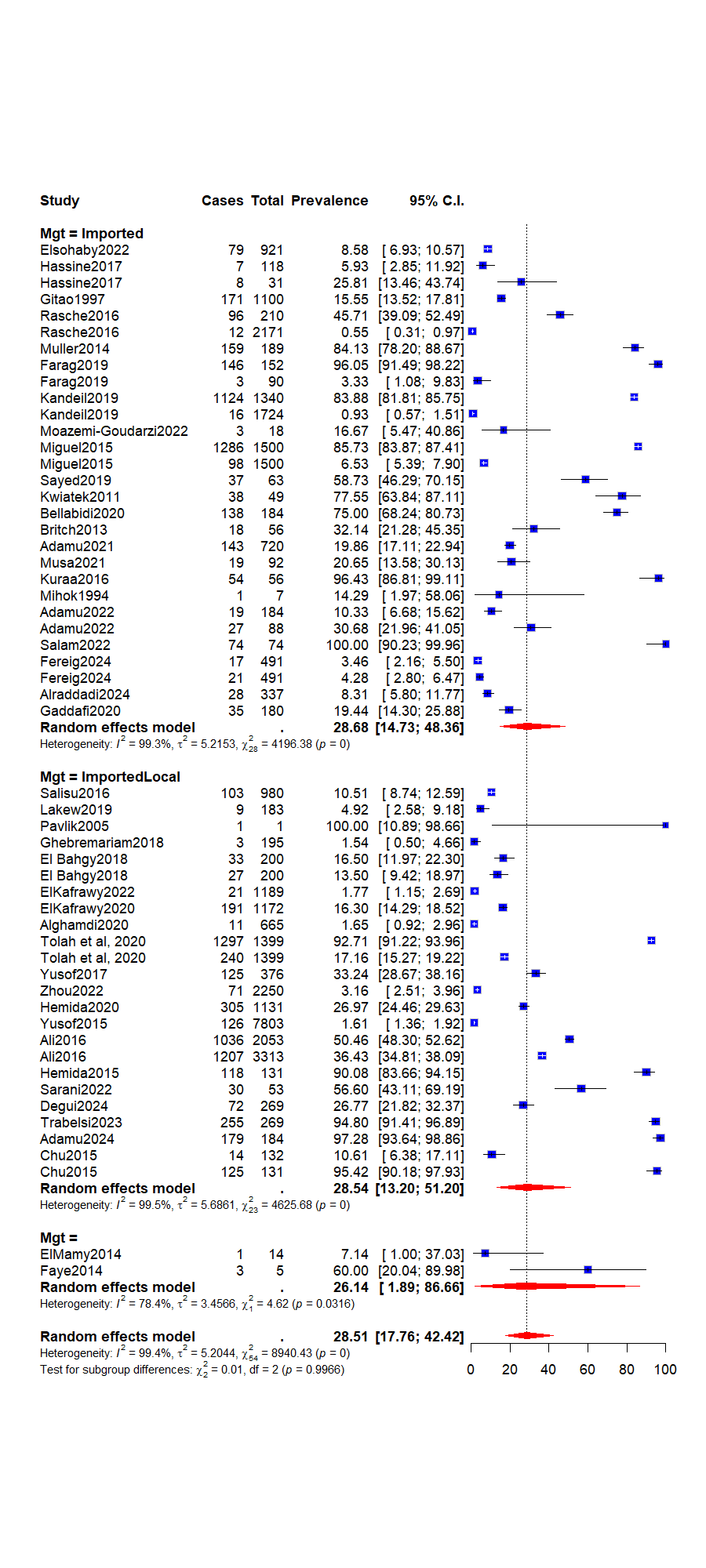


Figure S1: Forest plot for the subgroup analysis of TADs by management practice.


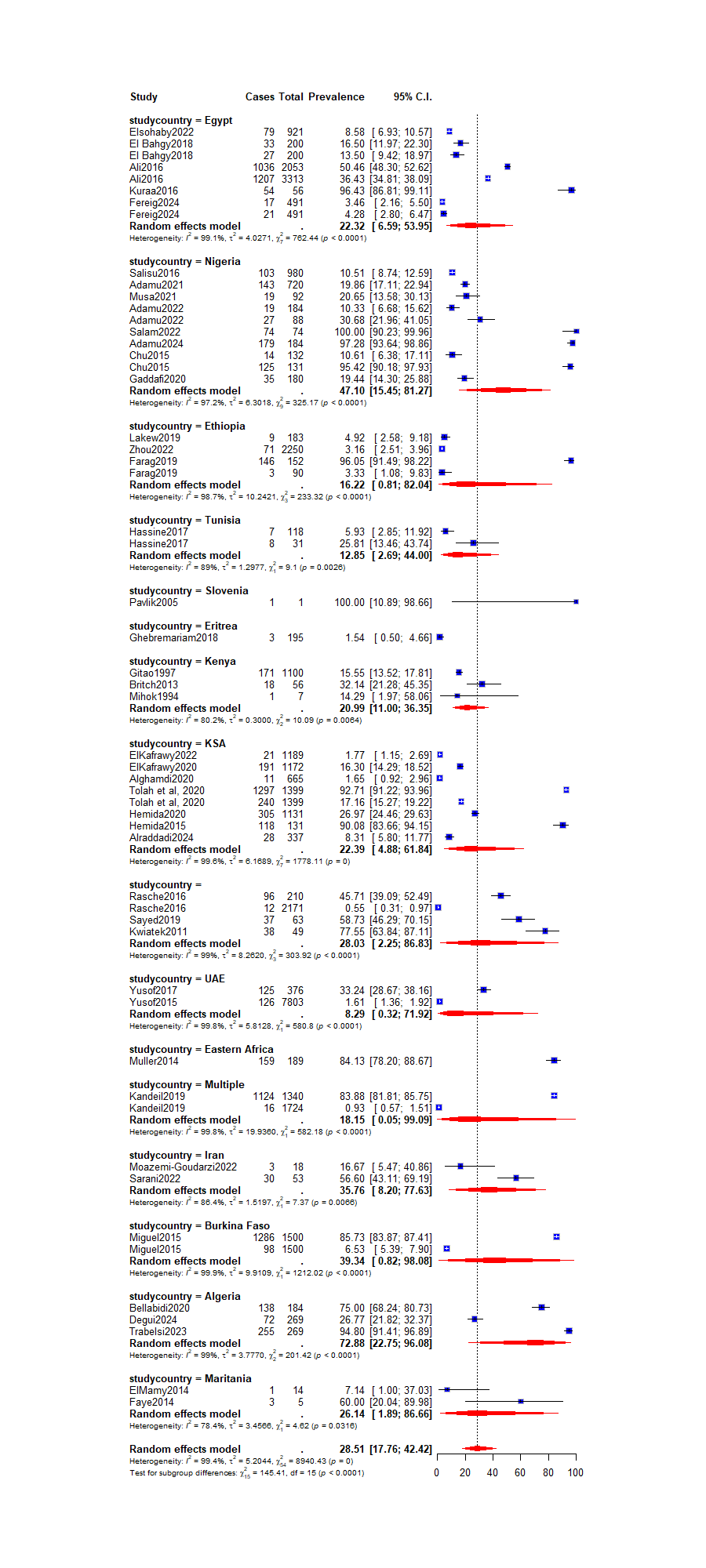


Figure S2: Forest plot for the subgroup analysis of TADs by location.


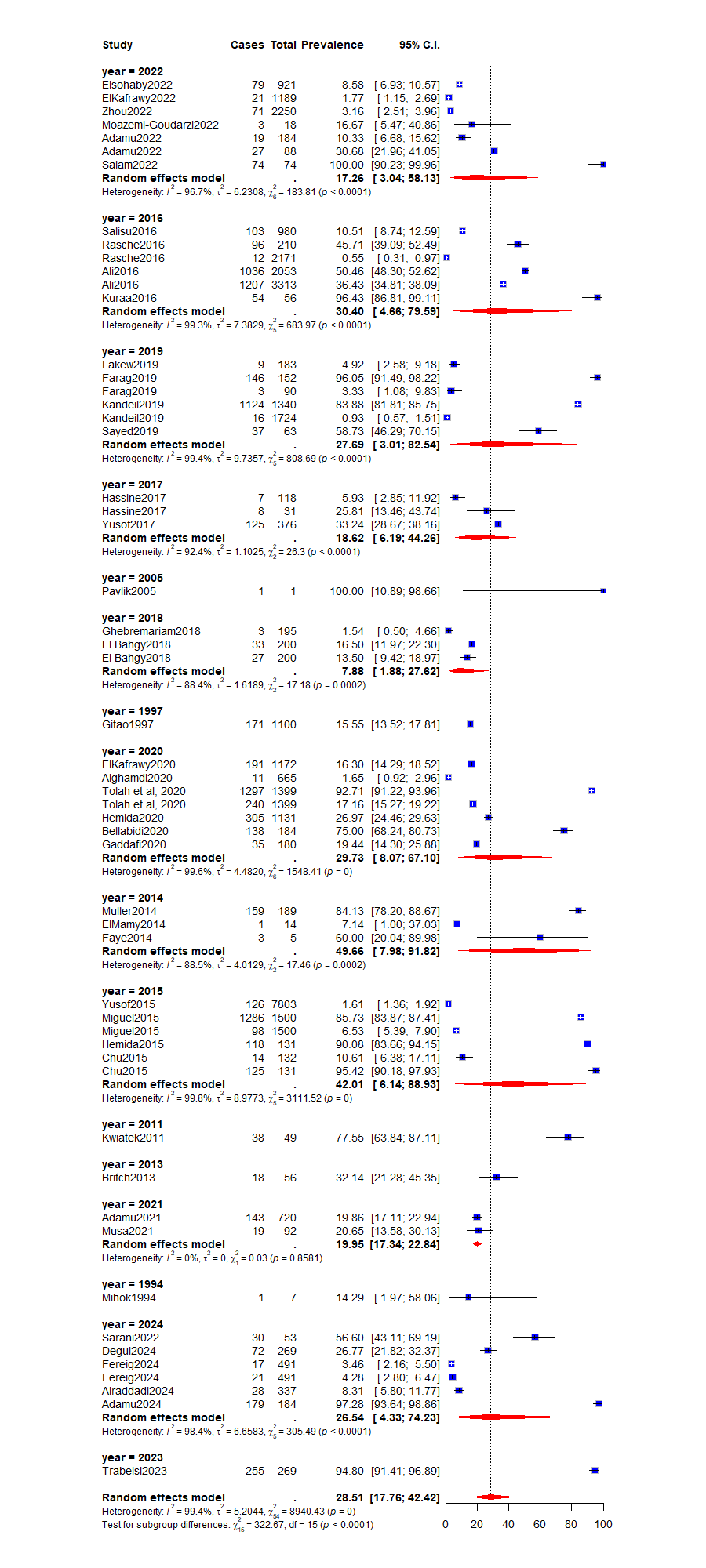


Figure S3: Forest plot for the subgroup analysis of TADs by research period.


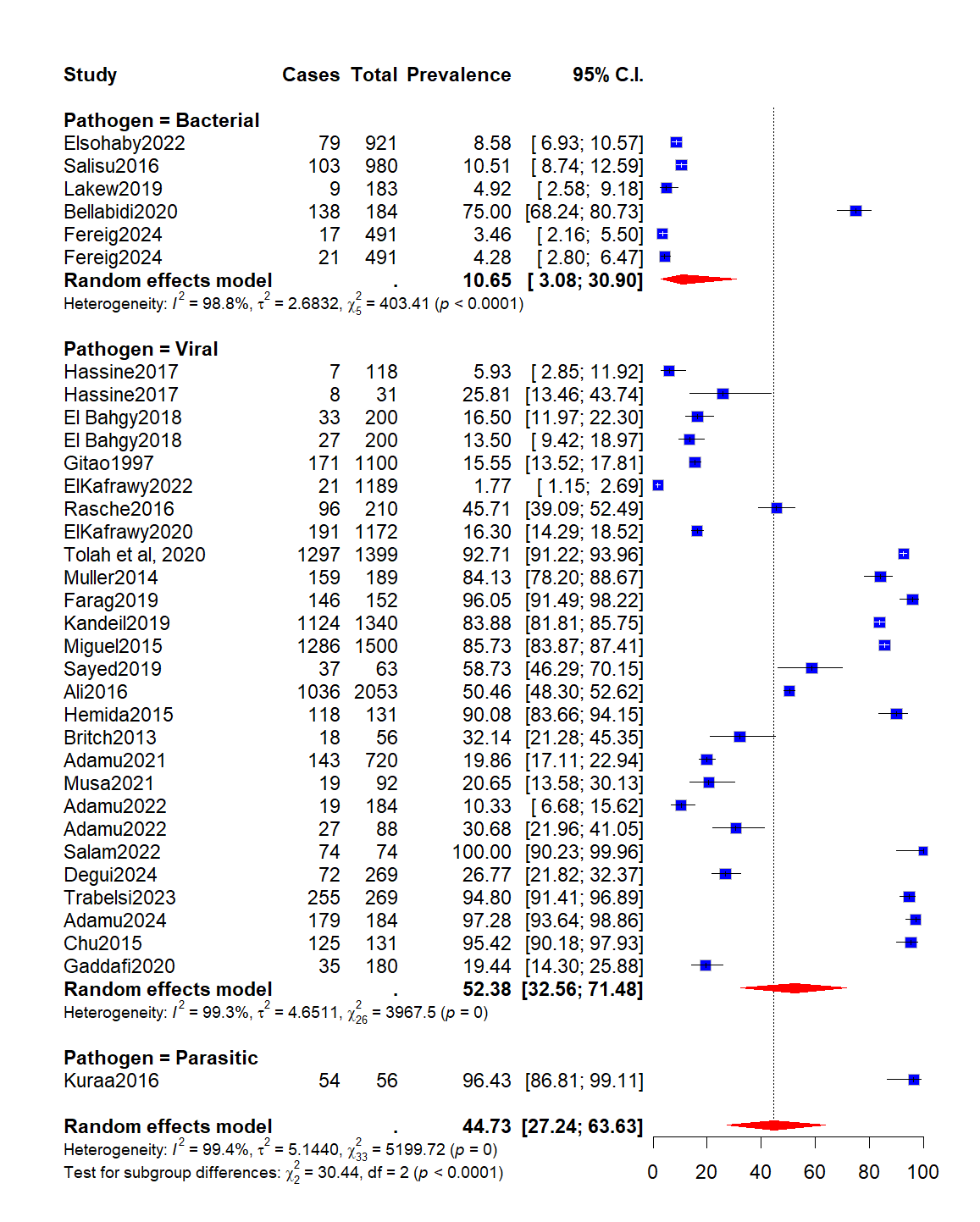


Figure S4: Forest plot for the subgroup analysis of TADs by pathogen.


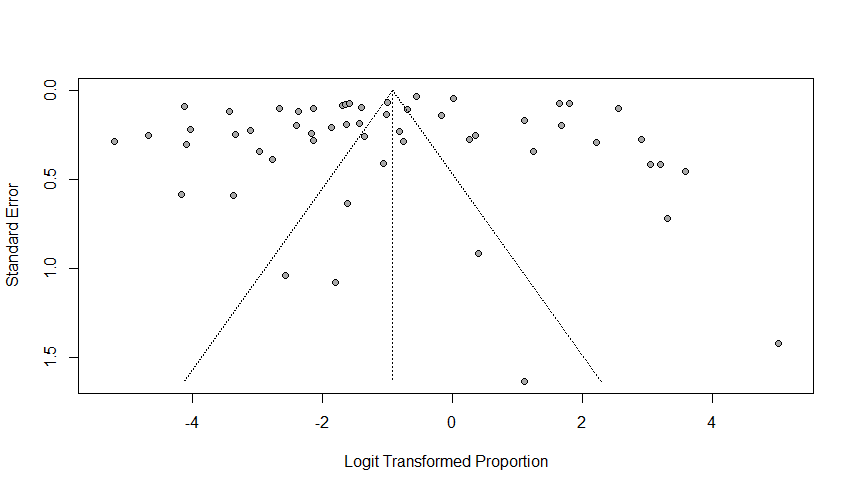


Figure S5: Funnel plot indicating publication bias in the overall prevalence of TADs.
